# Concomitant surgical cryoablation for refractory ventricular tachycardia and left ventricular assist device placement: a dual remedy but a recipe for thrombosis?

**DOI:** 10.1186/s13019-016-0451-x

**Published:** 2016-04-11

**Authors:** Colleen K. McIlvennan, Ashok N. Babu, Andreas Brieke, Amrut V. Ambardekar

**Affiliations:** Division of Cardiology, University of Colorado, School of Medicine, 12631 East 17th Avenue, B130, Aurora, CO 80045 Canada; Division of Cardiothoracic Surgery, University of Colorado, School of Medicine, Aurora, CO USA

**Keywords:** Heart-assist device, Heart failure, Ventricular tachycardia, Ablation

## Abstract

**Background:**

Ventricular tachycardia (VT) can persist following placement of a left ventricular assist device (LVAD). The optimal management strategy for VT during the peri-LVAD period is unknown.

**Case Presentations:**

Two case reports are presented that describe epicardial and endocardial VT ablation performed during LVAD placement. Subsequently, both patients developed LVAD thrombosis, a known and dreaded complication of LVADs, requiring re-operation.

**Conclusions:**

While LVAD thrombosis is likely multifactorial and remains an area of active research, these two cases should increase awareness of the possible risks of VT ablation—especially endocardial ablation—during LVAD placement. Further research is needed to understand the effects of VT ablation during the peri-LVAD period.

## Background

Left ventricular assist devices (LVADs) have the potential to improve survival and quality of life, yet complications are common—including ventricular tachycardia (VT) [1]. The ideal management strategy for VT peri-LVAD placement is unknown; however, there are isolated reports of performing concomitant surgical VT cryoablation at the time of LVAD placement with successful reduction in arrhythmia burden [2, 3]. In theory, there are advantages to performing such an open surgical ablation compared to percutaneous catheter based approaches including the ability to fully visualize the epicardial surface and left ventricular (LV) endocardium through the ventriculotomy that is required for LVAD inflow cannula placement. Herein, we present two cases of concomitant surgical epicardial and endocardial VT cryoablation prior to LVAD placement that resulted in successful treatment of VT, but were complicated by subsequent LVAD pump thrombosis (Table 1).

**Table 1:** Patient characteristics

	Patient #1	Patient #2
BASELINE		
Age	70	64
Gender	Female	Male
Etiology of Heart Failure	Non-Ischemic	Ischemic
VT Characteristics	Monomorphic at >188 bpm	Monomorphic at 135 bpm
Type of Surgical Ablation	Epicardial Endocardial	Epicardial Endocardial
LVAD	HeartMate II	HeartMate II
LVAD Indication	Destination Therapy	Bridge to Transplant
POST-LVAD		
Antiarrhythmic	Amiodarone	Amiodarone Mexiletine
Anticoagulation	Heparin Warfarin^a^	Heparin Warfarin^a^
Aspirin	81 mg	81 mg
Peak LDH^b^	2086 U/L	1368 U/L
Further VT	No	No
Thrombus Confirmed from Explanted LVAD	Yes	Yes

^a^International normalized ratio goal 2.0-3.0

^b^Normal lab value 124–271 U/L

*bpm* beats per minute, *LDH* lactate dehydrogenase, *LVAD* left ventricular assist device, *VT* ventricular tachycardia

## Case Presentation

### Case 1

A 70-year-old female with non-ischemic cardiomyopathy presented in cardiogenic shock with incessant monomorphic VT that required intravenous (IV) lidocaine and amiodarone to remain quiescent. Given her hemodynamics, an intra-aortic balloon pump was placed. She was stabilized over several days and was taken to the operating room where she underwent an open epicardial and endocardial VT ablation with cryoprobe. Pace activation mapping of the LV was performed until the patient’s morphologic VT was identified on the anterior lateral wall. Cryoablation at this site suppressed further VT development. Additional endocardial and epicardial ablation lines were performed to isolate the multiple areas of scar throughout the LV. This was followed by implantation of a HeartMate II (Thoratec, Pleasanton, CA) LVAD as destination therapy. Post-operatively, the patient was maintained on oral amiodarone, anticoagulated per guidelines with IV heparin, started on aspirin 81 mg daily and warfarin with a goal INR of 2.0–3.0. She had no sustained VT post-LVAD; however her lactate dehydrogenase (LDH) slowly rose to approximately two times the upper limit of normal. At this time, she had no obvious evidence of LVAD malfunction with a normal ramp echocardiogram and right heart catheterization confirming low left sided filling pressures and a cardiac output that matched the flow of the LVAD controller. Thus, she was subsequently discharged 4 weeks after implant. She presented 2 months later with an LDH seven times the upper limit of normal, hematuria, and heart failure symptoms. The patient underwent emergent device exchange for pump thrombosis and was discharged after a 3 week hospital stay. There was no evidence of thrombus in the LV cavity; however, examination of the explanted pump revealed a denatured thrombus ring adhered to the inlet bearing cup. Thirteen months later, she has not had any recurrence of sustained VT.

### Case 2

A 64-year-old male with ischemic cardiomyopathy was admitted with recurrent VT despite failing multiple oral antiarrhythmic medications. The patient was referred for percutaneous VT ablation; however, after induction of VT during the procedure, the patient arrested, required cardiopulmonary resuscitation, and was placed on veno-arterial extracorporeal membrane oxygenation. Intravenous amiodarone was initiated, and after several days of stabilization in the intensive care unit, the patient underwent open epicardial and endocardial VT ablation with cryoprobe. Pace activation mapping of the LV was performed and revealed that the patient’s morphologic VT came from an area near the LV septum, which was isolated with a circular cryoablation line. The patient’s VT was still inducible so several additional endocardial and epicardial ablation lines were required to terminate the patient’s VT. This was followed by implantation of a HeartMate II LVAD as bridge to transplant. Post-operatively, the patient was maintained on oral amiodarone and mexiletine with no further VT. He was anticoagulated per guidelines with IV heparin and received aspirin 81 mg daily and warfarin with a goal INR of 2.0–3.0. On post-implant day 18, the patient’s LDH started to slowly rise with a peak greater than five times the upper limit of normal. At post-implant day 20, he developed hematuria and acute renal failure and underwent emergent device exchange for device thrombosis. There was no evidence of thrombus in the LV cavity; however, there was confirmed thrombus on the pump rotor. The patient was discharged to acute rehab 4 weeks after device exchange. Nine months later, the patient is doing well and is free of any further VT.

## Discussion

In both cases, patients underwent open surgical cryoablation at the time of LVAD implantation and subsequently developed LVAD thrombosis. Cryoablation during LVAD implantation has been reported to decrease post-operative ventricular arrhythmias in select patients without an increase in the rate of adverse events [2–4]. However, several of these reports were published prior to the heightened awareness of thrombosis risk generated from more recent publications regarding LVAD pump thrombosis [5]. The etiology and risk factors for LVAD thrombosis remain an area of active investigation and is likely multifactorial.

While it is possible that other factors contributed to thrombosis in these patients, the likely association between the ablation procedures—specifically the LV endocardial ablation prior to LVAD inflow cannula placement—has a theoretical basis that deserves additional attention. First, ablation has been associated with an overall pro-inflammatory and thrombogenic state [6]. Second, it is also possible that the LV endocardial surface is more prone to thrombus formation related to inflammation and injury that results from the ablation itself. Finally, there are alterations in blood flow patterns within the LV related to flow into the LVAD inflow cannula that can result in low-flow stasis within certain portions of the LV cavity (e.g. due to the cannula position, the LV apex does not fully contract causing blood to pool). The combination of these three factors—thrombogenic state, endocardial inflammation, and stasis—are well known to be associated with thrombus formation [4]. Based on these factors, it may be reasonable to limit open surgical VT cryoablation to only epicardial ablation during LVAD placement.

## Conclusion

Given the substantial morbidity and risk for mortality with LVAD thrombosis and the continued uncertainty regarding mechanisms, these cases illustrate the complex patient factors that may contribute to this dreaded complication. Clinicians should be aware of the potential negative consequences of endocardial ablation at the time of LVAD placement. Further research (including the safety of isolated epicardial cryoablation without LV involvement) in the pre-operative management of VT in this population is warranted. In addition, larger multi-institutional LVAD studies are needed to confirm whether similar findings are present in a much broader cohort of patients.

## Consent

Written informed consent was obtained from the patients for publication of this case report and any accompanying images. A copy of the written consent is available for review by the Editor-in-Chief of this journal.

## Abbreviations

IV: intravenous
LDH: lactate dehydrogenase
LV: left ventricle
LVAD: left ventricular assist device
VT: ventricular tachycardia
